# Crystal structure of 2-*tert*-butyl-2,3-di­hydro-1*H*-benzo[*c*]pyrrol-1-one

**DOI:** 10.1107/S2056989017010337

**Published:** 2017-07-17

**Authors:** Joel Donkeng Dazie, Jiří Ludvík, Jan Fábry, Václav Eigner

**Affiliations:** a J. Heyrovsky Institute of Physical Chemistry, Academy of Sciences of the Czech Republic, Dolejškova 2155/3, 182 23 Prague 8, Czech Republic; b University of Chemistry and Technology, Technická 5, 166 28 Prague 6, Czech Republic; c Institute of Physics, Academy of Sciences of the Czech Republic, Na Slovance 2, 182 21 Praha 8, Czech Republic

**Keywords:** crystal structure, aromaticity, heterocyclic compounds, isoindolinone, angle strain, planarity

## Abstract

The asymmetric unit of the title compound, C_12_H_15_NO, comprises two sym­metry-independent mol­ecules which differ mainly in the conformations of the *tert*-butyl groups. The mol­ecules contain an essentially planar five-membered 3-pyrroline ring incorporating a carbonyl substituent (pyrrolinone) which forms part of an isoindolinone skeleton. The planarity of the pyrrole ring is compared to other structures with isoindolinone. There are only weak intra- and inter­molecular C—H⋯O and C—H⋯π-electron-ring inter­actions in the crystal structure.

## Abstract 2

The structure of 2-*tert*-butyl-2,3-di­hydro-1*H*-benzo[*c*]pyrrol-1-one is compared with those of the related compounds (3R*,1′S*,3′R*)-3-(1′-*tert*-butyl­amino-1′*H*,3′*H*-benzo[*c*]furan-3′-yl)-2-*tert*-butyl-2,3-di­hydro-1*H*-benzo[*c*]pyrrol-1-one and 2-isopropyl-2,3-di­hydro-1*H*-benzo[*c*]pyrrol-1-one, with special attention paid to the planarity of the substituted pyrrole rings in these structures.

## Chemical context   

**Figure 1:**
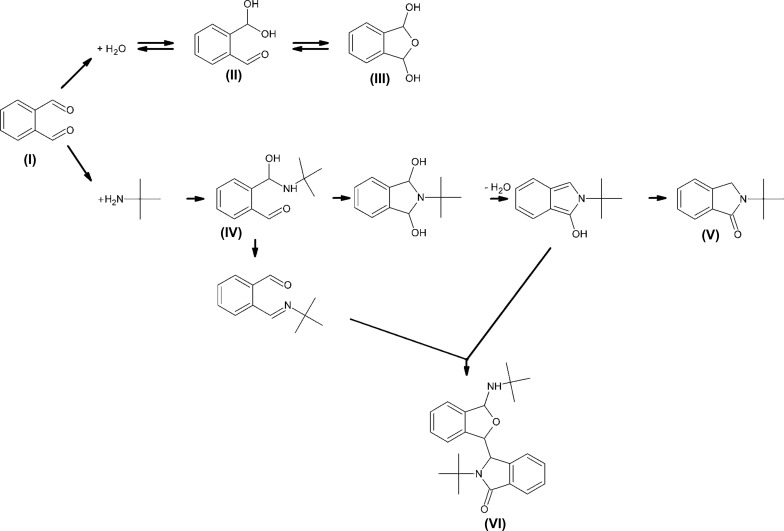
The reaction scheme for the synthesis of the title compound, (V).

Orthophthalaldehyde (*o*-phthalaldehyde, OPA) is an aromatic di­aldehyde bearing two electron-withdrawing carbonyl groups in positions 1 and 2. The reaction scheme involving OPA, (I), shown in Fig. 1 ▸ comprises the main concurrent as well as consecutive reactions, which are consistent with the results obtained herein. The reactions of OPA with primary amines, which were carried out by Winter (1900 ▸) and Thiele & Schneider (1909 ▸) for the first time, have been broadly applied for the synthesis of important heterocyclic compounds with biological relevance. A number of such reactions have been investigated recently and several structures of condensation products have been reported (DoMinh *et al.*, 1977 ▸; Nan’ya *et al.*, 1985 ▸; Takahashi *et al.*, 1996 ▸, 2004 ▸, 2005 ▸; Takahashi & Hatanaka, 1997 ▸). However, the reaction mechanism is still not fully understood. Determination of the products which would serve as a confirmation of the suggested reaction scheme (Fig. 1 ▸) is the reason for the present as well as for our previous studies (Donkeng Dazie, Liška & Ludvík, 2016 ▸; Donkeng Dazie, Liška, Ludvík, Fábry & Dušek, 2016 ▸; Donkeng Dazie *et al.*, 2017 ▸).
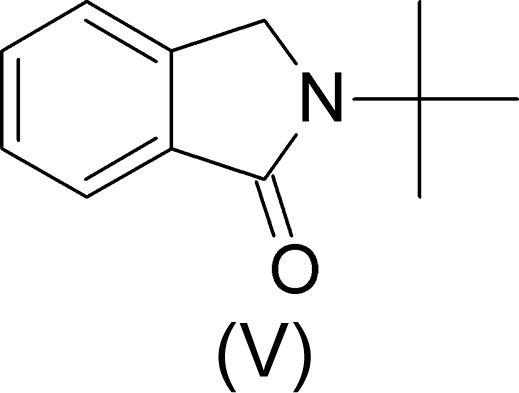


The reason why a full understanding of the reaction mechanism is still lacking is the complexity of the above-mentioned reactions, which are dependent on different variables. Our partly published electrochemical experiments have shown that the reaction kinetics, as well as the reaction products, depend on the primary amine which reacts with OPA, the reaction environment (solvent) and the proportion of the reactants (Donkeng Dazie, Liška & Ludvík, 2016 ▸). Electrochemical monitoring has indicated the presence of side reactions, which result in a mixture of mol­ecules of different mol­ecular weights with different proportions of OPA and primary amine building blocks [*cf.* the reaction of OPA with 2-amino­ethanol (kolamine); see Urban *et al.* (2007*a* ▸,*b* ▸)].

The complexity of the reactions between primary amines and OPA is affected by the environment in which they take place. The reaction of OPA with aliphatic primary amines in aqueous solutions involves competition between the amines and water mol­ecules as nucleophiles. Although water is a weaker nucleophile than primary amines, an enormous excess of water over primary amines may cause significant additional reactions, such as covalent hydration at the double bond of the carbonyl group and the following cyclization (Zuman, 2004 ▸) – see compounds (II) and (III) in Fig. 1 ▸. The reaction of OPA with aliphatic primary amines represents a concurrent process (DoMinh *et al.*, 1977 ▸). All attempts to isolate and identify the products of the reaction of OPA with primary amines in aqueous solutions were unsuccessful due to the number of reactions occurring and products, including the oligo- and polymeric ones (checked by thin-layer chromatography). In order to simplify the reaction media, diethyl ether as a non-aqueous organic solvent was used with the hope that some products might be obtained as crystals suitable for X-ray structure analysis.

Analogous to the reaction of OPA with iso­propyl­amine (Donkeng Dazie, Liška, Ludvík, Fábry & Dušek, 2016 ▸), the first step of the reaction with *tert*-butyl­amine results in a primary carbinolamine, (IV), the inter­mediate which further yields the title product, (V) (DoMinh *et al.*, 1977 ▸). The title product, (V), as well as co-product (VI), namely (3R*,1′S*,3′R*)-3-(1′-*tert*-butyl­amino-1′*H*,3′*H*-benzo[*c*]furan-3′-yl)-2-*tert*-butyl-2,3-di­hydro-1*H*-benzo[*c*]pyrrol-1-one) were identified as the main products in solution by means of ^1^H and ^13^C NMR analysis, as well as mass spectroscopy with electrospray ionization (ESI+). Compound (VI) was also crystallized and its structure has been determined previously (Donkeng Dazie *et al.*, 2017 ▸).

**Figure 2:**
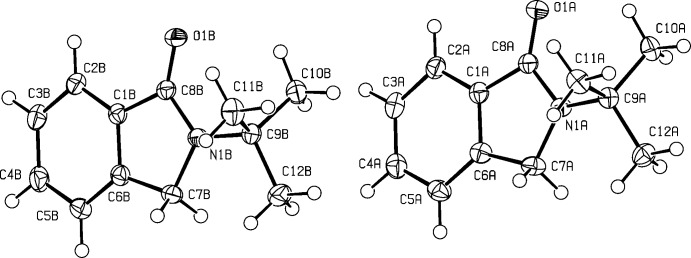
The atom-numbering scheme for the the two mol­ecules of (V) (*A* and *B*) in the asymmetric unit, with anisotropic displacement parameters shown at the 50% probability level.

The spectrometric results were confirmed unequivocally by the X-ray structure analysis of compound (V) (Fig. 2 ▸), as well as by the structure determination of (VI) (Donkeng Dazie *et al.*, 2017 ▸). In addition to the confirmation of the presence of the products in solution after they had been resolved as crystals, the previous crystallographic studies of (VI) and 2-isopropyl-2,3-di­hydro-1*H*-isoindol-1-one (Donkeng Dazie, Liška, Ludvík, Fábry & Dušek, 2016 ▸) were focused on the problem of planarity of the annelated pyrrole and furan rings.

The planarity of the pyrrole rings, which include two atoms close to *sp* ^3^-hybridized, was explained by propitious values of the inner angle in the regular penta­gon of 108°, *i.e.* close to the ideal tetra­hedral value of 109.54°. It turned out that the planarity is correlated on the C—N bond lengths in the pyrrole fragment. Specifically, pyrrole rings with longer N—C_carbon­yl_ bond lengths which exceed 1.39 Å tend to show better planarity than pyrrole rings with these shorter bond lengths (see Fig. 4 in the article by Donkeng Dazie, Liška, Ludvík, Fábry & Dušek, 2016 ▸).

The structure of (VI) (Donkeng Dazie *et al.*, 2017 ▸) contains pyrrole and furan rings as parts of isoindolinone and isobenzo­furan rings, respectively. The planarity of the pyrrole ring is extremely distorted in this structure and deviates more from planarity than the furan ring in the same structure. This phenomenon can be explained by steric reasons due to the presence of a voluminous *tert*-butyl group. The distortion of the pyrrole ring can be provoked by repulsion of the parts of the isoindolinone and isobenzo­furan rings which are close to each other. Therefore, the present structure determination is even more inter­esting because it offers a comparison of the distortion of the planarity of the pyrrole rings in the title structure with those in 2-*tert*-butyl-2,3-di­hydro-1*H*-benzo[*c*]pyrrol-1-one (Donkeng Dazie, Liška, Ludvík, Fábry & Dušek, 2016 ▸) and (3R*,1′S*,3′R*)-3-(1′-*tert*-butyl­amino-1′*H*,3′*H*-ben­zo[*c*]furan-3′-yl)-2-*tert*-butyl-2,3-di­hydro-1*H*-benzo[*c*]pyrrol-1-one, (VI) (Donkeng Dazie *et al.*, 2017 ▸), *i.e.* with respective less and more voluminous substituents.

## Synthesis and crystallization   

The synthesis of (V) was carried out at laboratory temperature under an argon atmosphere and the isolation procedure was similar to that reported by Takahashi *et al.* (2004 ▸, 2005 ▸). Orthophthalaldehyde (OPA, 0.335 g) was dissolved in diethyl ether (25 ml, 0.1 mol l^−1^) and *tert*-butyl­amine (0.183 g, 264 µl of the pure liquid compound) was added to the solution of OPA. The amounts of the reactants correspond to a 1:1 OPA–amine stoichiometric ratio. The reaction mixture was stirred for 6 h. The solution was filtered and the ether was evaporated under reduced pressure. Two previously mentioned compounds, *i.e.* (V) and (VI), were identified in a light-yellow oily solution by ^1^H and ^13^C NMR analysis, as well as mass spectroscopy. After a few days at room temperature, light-yellow crystals of (VI) of the size of several tenths of mm appeared. After half a year, other crystals appeared in the form of thin light-yellow needles which were as long as 2 cm. Their other dimensions were smaller than 0.1 mm. These crystals corresponded to the expected product, namely the title compound (V).

## Structural commentary   

**Figure 3:**
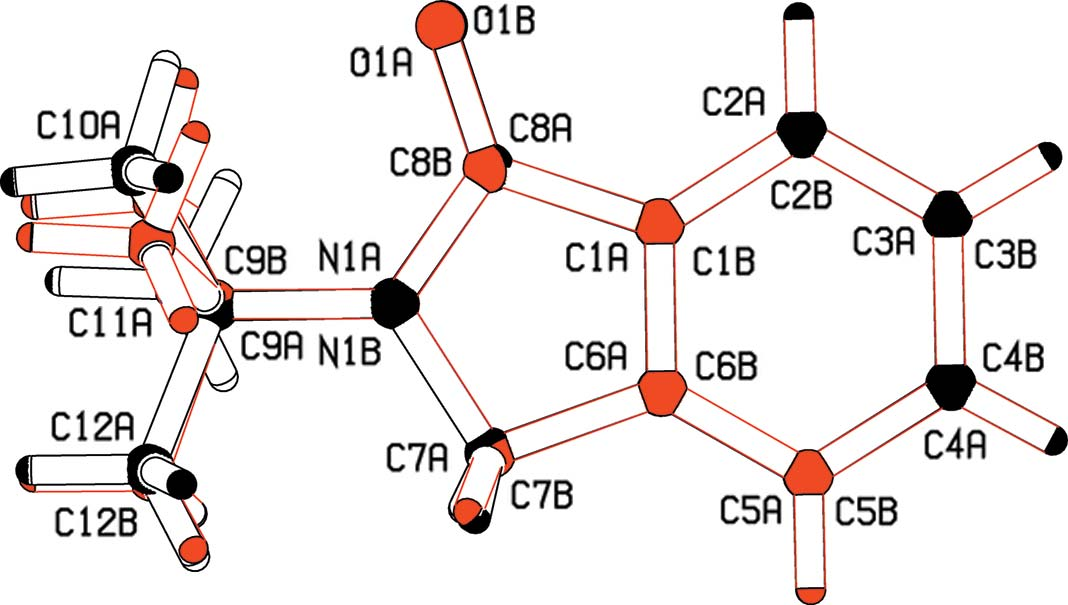
Two overlapped independent mol­ecules provided by *MolFit* in *PLATON* (Spek, 2009 ▸).

The title compound comprises two symmetry-independent mol­ecules (*A* and *B*) in the asymmetric unit (Fig. 2 ▸), the ring systems of which are approximately coplanar [dihedral angle between the planes = 8.38 (4)°]. The two mol­ecules are conformationally similar but not identical. The function *AutoMolFIT* in *PLATON* (Spek, 2009 ▸) yielded the weighted and unit-weight r.m.s. fits for the non-H atoms as 1.437 and 0.952 Å, respectively. The main difference between the two independent mol­ecules lies in the conformations within the *tert*-butyl substituent group (Fig. 3 ▸). These differences are reflected in the comparative values of the C7*A*/*B*—N1*A*/*B*—C9*A*/*B*—C10*A*/*B* torsion angles [151.25 (10) and 129.76 (11)°, respectively].

**Figure 4:**
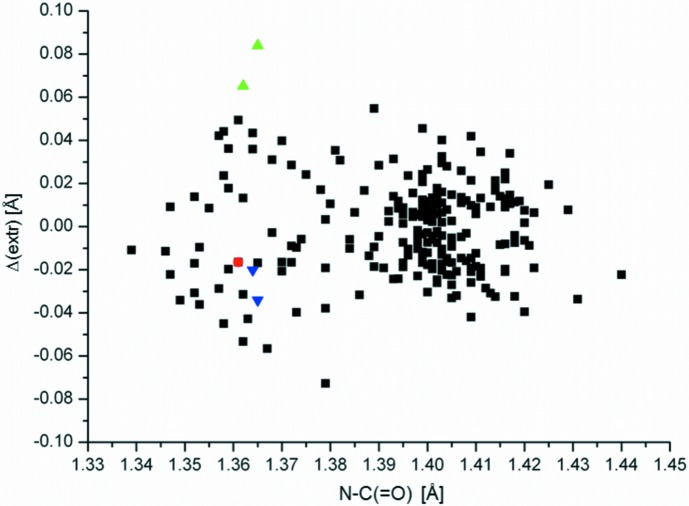
The dependence of the extremal deviations from planarity, Δ_extrem_ (Å), within the pyrrole core atoms of the isoindolinone system on the N—C bond length in N—C8(=O) (Å) (OriginLab, 2000 ▸). Black squares indicate the structures retrieved from the CSD, the red circle is 2-isopropyl-2,3-di­hydro-1*H*-isoindol-1-one (Donkeng Dazie, Liška, Ludvík, Fábry & Dušek, 2016 ▸), green triangles refer to (VI) (Donkeng Dazie *et al.*, 2017 ▸) and blue triangles refer to the title mol­ecule, (V).

Table 1 ▸ lists the extremal deviations from the fitted planes through the core atoms of the pyrrole rings in the title structure, *i.e.* without the carbonyl O atoms, which were omitted from considerations. Fig. 4 ▸ illustrates the dependence of the maximal deviations from the best plane through the core atoms of the pyrrole rings on the N—C_carbon­yl_ distance. Compounds include the title structure, (V), the structures determined by Donkeng Dazie, Liška, Ludvík, Fábry & Dušek (2016 ▸) and Donkeng Dazie *et al.* (2017 ▸), as well as 233 structures with the isoindolinone fragment (Table 1 ▸), which were retrieved from the Cambridge Structural Database (Version 5.36; Groom *et al.*, 2016 ▸). (The retrieved structures contained no disorder and errors, while they were determined below 150 K, with *R* factors < 0.05; in case the structures contained two carbonyl groups, the retrieved data were collected twice and the variant with the larger N—C8 distance was selected for further consideration.) Fig. 4 ▸ also shows that the largest distortion of the pyrrole ring takes place in (VI) (Donkeng Dazie *et al.*, 2017 ▸), the distortion being milder in the title mol­ecules and being mildest in 2-isopropyl-2,3-di­hydro-1*H*-isoindol-1-one (Donkeng Dazie, Liška, Ludvík, Fábry & Dušek, 2016 ▸). It indicates that the reasons for the distortion of the pyrrole rings from planarity are steric ones in these cases: *tert*-butyl as a more voluminous group causes a larger distortion in comparison with the isopropyl group. In (VI), an inter­action between the bulky isoindolinone and isobenzo­furan ring moieties also takes place.

## Supra­molecular features   

**Figure 5:**
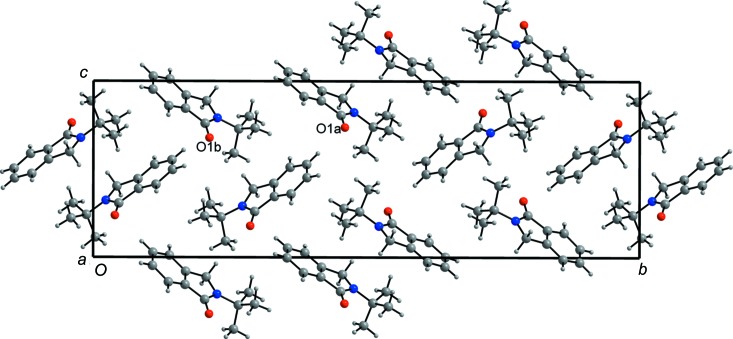
The packing of the title mol­ecules in the unit cell. H atoms have been omitted for clarity. Colour key: C gray, N blue and O red.

The crystal packing of the mol­ecules of (V) in the unit cell (Fig. 5 ▸) is relatively simple. There are two inter­molecular C—H⋯O inter­actions (Table 2 ▸), one linking the two independent mol­ecules (C4*B*—H⋯O1*A* ^ii^) and the other linking only *A* mol­ecules (C7*A*—H⋯O1*A* ^i^). Weak C—H⋯π-electron inter­actions involving only *B* mol­ecules (Table 3 ▸) are also present. No π–π-electron ring inter­actions are present in the structure.

## Database survey   

The survey relating particularly to the structural features of the isoindolinone ring system has been covered in §3.

## Refinement   

Crystal data, data collection and structure refinement details are summarized in Table 4 ▸. All H atoms were discernible in difference electron-density maps. However, the aryl, methyl­ene and methyl H atoms were constrained, with aryl C—H = 0.95 Å, methyl­ene C—H = 0.99 Å and methyl C—H = 0.98 Å, and with *U*_iso_(H) = 1.5*U*_eq_(C) for methyl H atoms and 1.2*U*_eq_(C) otherwise.

## Supplementary Material

Crystal structure: contains datablock(s) global, I. DOI: 10.1107/S2056989017010337/zs2383sup1.cif

Structure factors: contains datablock(s) I. DOI: 10.1107/S2056989017010337/zs2383Isup2.hkl

Supporting information file. DOI: 10.1107/S2056989017010337/zs2383Isup3.smi

Supporting information file. DOI: 10.1107/S2056989017010337/zs2383Isup4.cml

CCDC reference: 1561757

Additional supporting information:  crystallographic information; 3D view; checkCIF report

## Figures and Tables

**Table 1:** χ^2^ and extremal deviations (Å) from the fitted planes in the ring systems of the title mol­ecule

Ring	χ^2^	Extremal deviation (Å)	Atom with the greatest deviation
N1*A*—C1*A*—C2*A*—C3*A*—C4*A*—C5*A*—C6A—C7*A*—C8*A*	7649	0.0513 (10)	N1*A*
N1*B*—C1*B*—C2*B*—C3*B*—C4*B*—C5*B*—C6*B*—C7*B*—C8*B*	9338	0.0505 (10)	N1*B*
			
N1*A*—C1*A*—C6*A*—C7*A*—C8*A*	2590	−0.0341 (13)	C8A
N1*B*—C1*B*—C6*B*—C7*B*—C8*B*	923	−0.0201 (14)	C8B
			
C1*A*—C2*A*—C3*A*—C4*A*—C5*A*—C6*A*	160	0.0091 (14)	C3*A*
C1*B*—C2*B*—C3*B*—C4*B*—C5*B*—C6*B*	119	−0.0069 (14)	C5*B*

**Table 2:** Hydrogen-bond geometry (Å, °)

*D*—H⋯*A*	*D*—H	H⋯*A*	*D*⋯*A*	*D*—H⋯*A*
C7*A*—H2c7*A*⋯O1*A* ^i^	0.99	2.51	3.4389 (14)	157
C10*A*—H1c10*A*⋯O1*A*	0.98	2.34	2.9149 (12)	117
C4*B*—H1c4*B*⋯O1*A* ^ii^	0.95	2.39	3.3237 (15)	167
C10*B*—H2c1*B*⋯O1*B*	0.98	2.42	3.0171 (15)	119

Symmetry codes: (i) 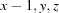; (ii) 
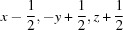
.

**Table 3:** C—H⋯π-electron ring inter­actions (Å, °) *Cg*1 is the centroid of the N1*B*/C7*B*/C6*B*/C1*B*/C8*B* ring and *Cg*2 is the centroid of the C1*B*/C2*B*/C3*B*/C4*B*/C5*B*/C6*B* ring.

C—H⋯*Cg*	C—H	H⋯*Cg*	C—H⋯*Cg*	C⋯*Cg*
C11*B*—H3c11*B*⋯*Cg*1^iii^	0.98	2.78	135	3.5373 (14)
C11*B*—H3c11*B*⋯*Cg*2^iii^	0.98	2.95	173	3.9288 (14)

Symmetry code: (iii): *x* − 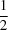, −*y* + 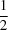, *z* − 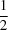.

**Table 4:** Experimental details

Crystal data	
Chemical formula	C_12_H_15_NO
*M*_r_	189.3
Crystal system, space group	Monoclinic, *P*2_1_/*n*
Temperature (K)	120
*a*, *b*, *c* (Å)	6.0440 (1), 32.6938 (6), 10.5679 (2)
β (°)	92.266 (2)
*V* (Å^3^)	2086.60 (7)
*Z*	8
Radiation type	Cu *K*α
μ (mm^−1^)	0.60
Crystal size (mm)	0.71 × 0.05 × 0.04

Data collection	
Diffractometer	Rigaku Xcalibur (Atlas S2, Gemini ultra)
Absorption correction	Analytical (*CrysAlis PRO*; Rigaku OD, 2015 ▸)
*T*_min_, *T*_max_	0.912, 0.990
No. of measured, independent and observed [*I* > 3σ(*I*)] reflections	14273, 3683, 3183
*R*_int_	0.022
(sin θ/λ)_max_ (Å^−1^)	0.597

Refinement	
*R*[*F* > 3σ(*F*)], *wR*(*F*), *S*	0.032, 0.083, 2.13
No. of reflections	3683
No. of parameters	254
H-atom treatment	H-atom parameters constrained
Δρ_max_, Δρ_min_ (e Å^−3^)	0.21, −0.15

Computer programs: *CrysAlis PRO* (Rigaku OD, 2015 ▸), *SIR2014* (Burla *et al.*, 2015 ▸), *JANA2006* (Petříček *et al.*, 2014 ▸), *DIAMOND* (Brandenburg & Putz, 2005 ▸), *Origin6.1* (OriginLab, 2000 ▸) and *PLATON* (Spek, 2009 ▸).

## supplementary crystallographic information

### Crystal data

**Table:**

C_12_H_15_NO	*F*(000) = 816
*M**_r_* = 189.3	*D*_x_ = 1.205 Mg m^−^^3^
Monoclinic, *P*2_1_/*n*	Cu *K*α radiation, λ = 1.54184 Å
Hall symbol: -P 2yn	Cell parameters from 7498 reflections
*a* = 6.0440 (1) Å	θ = 4.4–67.0°
*b* = 32.6938 (6) Å	µ = 0.60 mm^−^^1^
*c* = 10.5679 (2) Å	*T* = 120 K
β = 92.266 (2)°	Needle, yellow
*V* = 2086.60 (7) Å^3^	0.71 × 0.05 × 0.04 mm
*Z* = 8	

### Data collection

**Table:**

Rigaku Xcalibur (AtlasS2, Gemini ultra) diffractometer	3683 independent reflections
Radiation source: fine-focus sealed X-ray tube	3183 reflections with *I* > 3σ(*I*)
Mirror monochromator	*R*_int_ = 0.022
Detector resolution: 5.1783 pixels mm^-1^	θ_max_ = 67.0°, θ_min_ = 4.2°
ω scans	*h* = −7→4
Absorption correction: analytical (CrysAlis PRO; Rigaku OD, 2015)	*k* = −38→38
*T*_min_ = 0.912, *T*_max_ = 0.990	*l* = −12→12
14273 measured reflections	

### Refinement

**Table:**

Refinement on *F*^2^	H-atom parameters constrained
*R*[*F* > 3σ(*F*)] = 0.032	Weighting scheme based on measured s.u.'s *w* = 1/(σ^2^(*I*) + 0.0004*I*^2^)
*wR*(*F*) = 0.083	(Δ/σ)_max_ = 0.014
*S* = 2.13	Δρ_max_ = 0.21 e Å^−^^3^
3683 reflections	Δρ_min_ = −0.15 e Å^−^^3^
254 parameters	Extinction correction: B-C type 1 Lorentzian isotropic (Becker & Coppens, 1974)
0 restraints	Extinction coefficient: 1900 (300)
120 constraints	

### Fractional atomic coordinates and isotropic or equivalent isotropic displacement parameters (Å^2^)

**Table:**

	*x*	*y*	*z*	*U*_iso_*/*U*_eq_	
C1A	1.05872 (18)	0.41691 (3)	0.88936 (11)	0.0210 (3)	
C2A	1.20864 (19)	0.38519 (3)	0.91362 (11)	0.0248 (3)	
H1c2A	1.346861	0.384309	0.873912	0.0297*	
C3A	1.1490 (2)	0.35501 (4)	0.99761 (12)	0.0280 (4)	
H1c3A	1.249274	0.333402	1.01793	0.0336*	
C4A	0.9431 (2)	0.35599 (4)	1.05272 (12)	0.0284 (4)	
H1c4A	0.904144	0.334728	1.108911	0.0341*	
C5A	0.7941 (2)	0.38750 (4)	1.02689 (11)	0.0272 (4)	
H1c5A	0.653838	0.388023	1.06438	0.0326*	
C6A	0.85583 (18)	0.41824 (4)	0.94471 (11)	0.0225 (3)	
C7A	0.73755 (19)	0.45661 (4)	0.90290 (11)	0.0248 (3)	
H1c7A	0.710928	0.473925	0.977636	0.0298*	
H2c7A	0.603511	0.449372	0.850811	0.0298*	
N1A	0.89842 (15)	0.47684 (3)	0.82295 (9)	0.0202 (3)	
C8A	1.08131 (18)	0.45317 (3)	0.80689 (10)	0.0201 (3)	
O1A	1.23619 (13)	0.46031 (2)	0.73771 (8)	0.0271 (3)	
C9A	0.83129 (17)	0.51233 (3)	0.74110 (10)	0.0207 (3)	
C10A	1.02829 (19)	0.54069 (3)	0.72184 (12)	0.0260 (3)	
H1c10A	1.138881	0.526448	0.672735	0.0389*	
H2c10A	1.094491	0.548562	0.804426	0.0389*	
H3c10A	0.977748	0.565243	0.676015	0.0389*	
C11A	0.7424 (2)	0.49567 (4)	0.61388 (11)	0.0266 (4)	
H1c11A	0.610011	0.479131	0.626943	0.04*	
H2c11A	0.704256	0.518517	0.557074	0.04*	
H3c11A	0.855888	0.47869	0.576052	0.04*	
C12A	0.6517 (2)	0.53639 (4)	0.80661 (12)	0.0281 (4)	
H1c12A	0.707293	0.544869	0.890941	0.0421*	
H2c12A	0.611984	0.560649	0.756188	0.0421*	
H3c12A	0.520623	0.519082	0.814695	0.0421*	
C1B	0.93503 (18)	0.16836 (3)	0.84550 (11)	0.0200 (3)	
C2B	1.0953 (2)	0.13796 (3)	0.86011 (11)	0.0240 (3)	
H1c2B	1.22006	0.137301	0.808191	0.0288*	
C3B	1.0668 (2)	0.10871 (4)	0.95295 (12)	0.0288 (4)	
H1c3B	1.175008	0.087889	0.96595	0.0346*	
C4B	0.8815 (2)	0.10939 (4)	1.02764 (12)	0.0307 (4)	
H1c4B	0.864377	0.088894	1.090217	0.0368*	
C5B	0.7219 (2)	0.13970 (4)	1.01152 (12)	0.0285 (4)	
H1c5B	0.594989	0.140041	1.061777	0.0343*	
C6B	0.75219 (19)	0.16947 (3)	0.92019 (11)	0.0222 (3)	
C7B	0.61887 (19)	0.20715 (3)	0.88797 (11)	0.0231 (3)	
H1c7B	0.472526	0.199046	0.850699	0.0278*	
H2c7B	0.614963	0.225101	0.963283	0.0278*	
N1B	0.74871 (15)	0.22684 (3)	0.79078 (9)	0.0203 (3)	
C8B	0.92917 (18)	0.20442 (3)	0.76015 (10)	0.0197 (3)	
O1B	1.06256 (13)	0.21250 (2)	0.67857 (8)	0.0265 (2)	
C9B	0.68069 (18)	0.26616 (3)	0.72888 (11)	0.0216 (3)	
C10B	0.8756 (2)	0.29586 (4)	0.73421 (13)	0.0298 (4)	
H1c10B	0.93154	0.298755	0.822062	0.0447*	
H2c10B	0.993636	0.285303	0.682254	0.0447*	
H3c10B	0.826834	0.322588	0.70173	0.0447*	
C11B	0.6048 (2)	0.25745 (4)	0.59210 (12)	0.0305 (4)	
H1c11B	0.473875	0.23976	0.59104	0.0458*	
H2c11B	0.724073	0.243737	0.548356	0.0458*	
H3c11B	0.567854	0.283251	0.549073	0.0458*	
C12B	0.4915 (2)	0.28510 (4)	0.80033 (13)	0.0317 (4)	
H1c12B	0.36695	0.265921	0.800773	0.0475*	
H2c12B	0.443891	0.310544	0.758456	0.0475*	
H3c12B	0.542212	0.291011	0.887656	0.0475*	

### Atomic displacement parameters (Å^2^)

**Table:**

	*U*^11^	*U*^22^	*U*^33^	*U*^12^	*U*^13^	*U*^23^
C1A	0.0229 (6)	0.0211 (6)	0.0187 (5)	−0.0019 (4)	−0.0019 (4)	−0.0036 (4)
C2A	0.0254 (6)	0.0213 (6)	0.0277 (6)	0.0009 (4)	0.0009 (5)	−0.0042 (5)
C3A	0.0361 (7)	0.0201 (6)	0.0275 (6)	0.0035 (5)	−0.0020 (5)	−0.0025 (5)
C4A	0.0404 (7)	0.0231 (6)	0.0216 (6)	−0.0022 (5)	0.0010 (5)	0.0004 (5)
C5A	0.0294 (6)	0.0306 (6)	0.0218 (6)	−0.0031 (5)	0.0038 (5)	0.0015 (5)
C6A	0.0234 (6)	0.0263 (6)	0.0176 (5)	−0.0012 (4)	−0.0009 (4)	−0.0006 (5)
C7A	0.0210 (6)	0.0316 (6)	0.0219 (6)	0.0013 (4)	0.0035 (5)	0.0052 (5)
N1A	0.0193 (5)	0.0228 (5)	0.0183 (5)	0.0012 (3)	0.0008 (4)	0.0013 (4)
C8A	0.0199 (5)	0.0214 (5)	0.0190 (5)	−0.0023 (4)	−0.0005 (4)	−0.0037 (4)
O1A	0.0236 (4)	0.0262 (4)	0.0320 (5)	−0.0007 (3)	0.0091 (4)	0.0012 (4)
C9A	0.0218 (5)	0.0216 (6)	0.0186 (5)	0.0009 (4)	−0.0019 (4)	0.0017 (4)
C10A	0.0261 (6)	0.0221 (6)	0.0293 (6)	−0.0033 (4)	−0.0032 (5)	0.0016 (5)
C11A	0.0289 (6)	0.0299 (6)	0.0207 (6)	−0.0049 (5)	−0.0044 (5)	0.0013 (5)
C12A	0.0287 (6)	0.0278 (6)	0.0277 (6)	0.0068 (5)	0.0004 (5)	0.0008 (5)
C1B	0.0221 (5)	0.0188 (5)	0.0189 (5)	−0.0022 (4)	−0.0010 (4)	−0.0032 (4)
C2B	0.0262 (6)	0.0212 (6)	0.0248 (6)	0.0014 (4)	0.0022 (5)	−0.0037 (5)
C3B	0.0369 (7)	0.0200 (6)	0.0295 (7)	0.0053 (5)	0.0004 (5)	−0.0011 (5)
C4B	0.0463 (7)	0.0196 (6)	0.0264 (6)	−0.0007 (5)	0.0048 (5)	0.0027 (5)
C5B	0.0343 (7)	0.0249 (6)	0.0271 (6)	−0.0030 (5)	0.0095 (5)	0.0013 (5)
C6B	0.0242 (6)	0.0204 (6)	0.0220 (6)	−0.0027 (4)	0.0010 (5)	−0.0023 (4)
C7B	0.0213 (5)	0.0242 (6)	0.0242 (6)	−0.0006 (4)	0.0051 (5)	0.0026 (5)
N1B	0.0193 (4)	0.0212 (5)	0.0206 (5)	0.0019 (4)	0.0028 (4)	0.0022 (4)
C8B	0.0198 (5)	0.0204 (5)	0.0189 (5)	−0.0011 (4)	0.0002 (4)	−0.0032 (4)
O1B	0.0270 (4)	0.0262 (4)	0.0271 (4)	0.0023 (3)	0.0107 (3)	0.0028 (3)
C9B	0.0220 (6)	0.0195 (5)	0.0233 (6)	0.0023 (4)	0.0009 (4)	0.0024 (4)
C10B	0.0278 (6)	0.0227 (6)	0.0390 (7)	−0.0016 (5)	0.0028 (5)	−0.0005 (5)
C11B	0.0360 (7)	0.0282 (7)	0.0268 (7)	0.0020 (5)	−0.0061 (5)	0.0040 (5)
C12B	0.0292 (6)	0.0284 (6)	0.0379 (7)	0.0088 (5)	0.0081 (5)	0.0047 (5)

### Geometric parameters (Å, º)

**Table:**

C1A—C2A	1.3942 (16)	C1B—C2B	1.3922 (16)
C1A—C6A	1.3800 (16)	C1B—C6B	1.3836 (16)
C1A—C8A	1.4810 (16)	C1B—C8B	1.4841 (15)
C2A—H1c2A	0.95	C2B—H1c2B	0.95
C2A—C3A	1.3844 (17)	C2B—C3B	1.3858 (17)
C3A—H1c3A	0.95	C3B—H1c3B	0.95
C3A—C4A	1.3949 (18)	C3B—C4B	1.3958 (19)
C4A—H1c4A	0.95	C4B—H1c4B	0.95
C4A—C5A	1.3881 (17)	C4B—C5B	1.3887 (18)
C5A—H1c5A	0.95	C5B—H1c5B	0.95
C5A—C6A	1.3889 (17)	C5B—C6B	1.3880 (17)
C6A—C7A	1.5018 (16)	C6B—C7B	1.5036 (16)
C7A—H1c7A	0.99	C7B—H1c7B	0.99
C7A—H2c7A	0.99	C7B—H2c7B	0.99
C7A—N1A	1.4703 (15)	C7B—N1B	1.4659 (15)
H1c7A—H2c7A	1.6713	N1B—C8B	1.3636 (14)
N1A—C8A	1.3655 (14)	N1B—C9B	1.4929 (14)
N1A—C9A	1.4936 (14)	C8B—O1B	1.2320 (14)
C8A—O1A	1.2326 (14)	C9B—C10B	1.5259 (16)
C9A—C10A	1.5291 (16)	C9B—C11B	1.5259 (17)
C9A—C11A	1.5283 (16)	C9B—C12B	1.5262 (17)
C9A—C12A	1.5283 (16)	C10B—H1c10B	0.98
C10A—H1c10A	0.98	C10B—H2c10B	0.98
C10A—H2c10A	0.98	C10B—H3c10B	0.98
C10A—H3c10A	0.98	C11B—H1c11B	0.98
C11A—H1c11A	0.98	C11B—H2c11B	0.98
C11A—H2c11A	0.98	C11B—H3c11B	0.98
C11A—H3c11A	0.98	C12B—H1c12B	0.98
C12A—H1c12A	0.98	C12B—H2c12B	0.98
C12A—H2c12A	0.98	C12B—H3c12B	0.98
C12A—H3c12A	0.98		
			
C2A—C1A—C6A	121.84 (11)	C2B—C1B—C6B	121.57 (10)
C2A—C1A—C8A	129.00 (10)	C2B—C1B—C8B	129.36 (10)
C6A—C1A—C8A	109.16 (10)	C6B—C1B—C8B	108.99 (9)
C1A—C2A—H1c2A	121.18	C1B—C2B—H1c2B	121.12
C1A—C2A—C3A	117.65 (11)	C1B—C2B—C3B	117.76 (11)
H1c2A—C2A—C3A	121.18	H1c2B—C2B—C3B	121.12
C2A—C3A—H1c3A	119.65	C2B—C3B—H1c3B	119.52
C2A—C3A—C4A	120.70 (11)	C2B—C3B—C4B	120.95 (11)
H1c3A—C3A—C4A	119.65	H1c3B—C3B—C4B	119.52
C3A—C4A—H1c4A	119.42	C3B—C4B—H1c4B	119.63
C3A—C4A—C5A	121.16 (11)	C3B—C4B—C5B	120.75 (11)
H1c4A—C4A—C5A	119.42	H1c4B—C4B—C5B	119.63
C4A—C5A—H1c5A	120.93	C4B—C5B—H1c5B	120.81
C4A—C5A—C6A	118.14 (11)	C4B—C5B—C6B	118.39 (12)
H1c5A—C5A—C6A	120.93	H1c5B—C5B—C6B	120.81
C1A—C6A—C5A	120.49 (11)	C1B—C6B—C5B	120.57 (11)
C1A—C6A—C7A	108.80 (10)	C1B—C6B—C7B	108.98 (10)
C5A—C6A—C7A	130.69 (11)	C5B—C6B—C7B	130.36 (11)
C6A—C7A—H1c7A	109.47	C6B—C7B—H1c7B	109.47
C6A—C7A—H2c7A	109.47	C6B—C7B—H2c7B	109.47
C6A—C7A—N1A	103.12 (9)	C6B—C7B—N1B	102.80 (9)
H1c7A—C7A—H2c7A	115.15	H1c7B—C7B—H2c7B	115.41
H1c7A—C7A—N1A	109.47	H1c7B—C7B—N1B	109.47
H2c7A—C7A—N1A	109.47	H2c7B—C7B—N1B	109.47
C7A—N1A—C8A	111.93 (9)	C7B—N1B—C8B	112.69 (9)
C7A—N1A—C9A	120.76 (8)	C7B—N1B—C9B	122.65 (9)
C8A—N1A—C9A	124.82 (9)	C8B—N1B—C9B	124.60 (9)
C1A—C8A—N1A	106.66 (9)	C1B—C8B—N1B	106.42 (9)
C1A—C8A—O1A	126.18 (10)	C1B—C8B—O1B	126.51 (10)
N1A—C8A—O1A	127.15 (10)	N1B—C8B—O1B	127.06 (10)
N1A—C9A—C10A	110.67 (9)	N1B—C9B—C10B	109.45 (9)
N1A—C9A—C11A	108.10 (9)	N1B—C9B—C11B	108.75 (9)
N1A—C9A—C12A	108.61 (9)	N1B—C9B—C12B	109.35 (9)
C10A—C9A—C11A	110.33 (9)	C10B—C9B—C11B	110.88 (10)
C10A—C9A—C12A	108.60 (9)	C10B—C9B—C12B	108.42 (9)
C11A—C9A—C12A	110.52 (9)	C11B—C9B—C12B	109.97 (9)
C9A—C10A—H1c10A	109.47	C9B—C10B—H1c10B	109.47
C9A—C10A—H2c10A	109.47	C9B—C10B—H2c10B	109.47
C9A—C10A—H3c10A	109.47	C9B—C10B—H3c10B	109.47
H1c10A—C10A—H2c10A	109.47	H1c10B—C10B—H2c10B	109.47
H1c10A—C10A—H3c10A	109.47	H1c10B—C10B—H3c10B	109.47
H2c10A—C10A—H3c10A	109.47	H2c10B—C10B—H3c10B	109.47
C9A—C11A—H1c11A	109.47	C9B—C11B—H1c11B	109.47
C9A—C11A—H2c11A	109.47	C9B—C11B—H2c11B	109.47
C9A—C11A—H3c11A	109.47	C9B—C11B—H3c11B	109.47
H1c11A—C11A—H2c11A	109.47	H1c11B—C11B—H2c11B	109.47
H1c11A—C11A—H3c11A	109.47	H1c11B—C11B—H3c11B	109.47
H2c11A—C11A—H3c11A	109.47	H2c11B—C11B—H3c11B	109.47
C9A—C12A—H1c12A	109.47	C9B—C12B—H1c12B	109.47
C9A—C12A—H2c12A	109.47	C9B—C12B—H2c12B	109.47
C9A—C12A—H3c12A	109.47	C9B—C12B—H3c12B	109.47
H1c12A—C12A—H2c12A	109.47	H1c12B—C12B—H2c12B	109.47
H1c12A—C12A—H3c12A	109.47	H1c12B—C12B—H3c12B	109.47
H2c12A—C12A—H3c12A	109.47	H2c12B—C12B—H3c12B	109.47
			
C9A—N1A—C8A—C1A	−168.13 (9)	C9B—N1B—C8B—C1B	179.36 (9)
C7A—N1A—C9A—C10A	151.25 (10)	C7B—N1B—C9B—C10B	129.76 (11)
C8A—N1A—C7A—C6A	5.02 (12)	C8B—N1B—C7B—C6B	3.02 (12)
C9A—N1A—C7A—C6A	167.96 (9)	C9B—N1B—C7B—C6B	−179.88 (9)
C7A—N1A—C8A—O1A	174.11 (11)	C7B—N1B—C8B—O1B	176.92 (10)
C9A—N1A—C8A—O1A	11.99 (17)	C9B—N1B—C8B—O1B	−0.12 (17)
C7A—N1A—C8A—C1A	−6.01 (12)	C7B—N1B—C8B—C1B	−3.60 (12)
C7A—N1A—C9A—C11A	−87.82 (12)	C7B—N1B—C9B—C11B	−108.96 (11)
C8A—N1A—C9A—C11A	72.82 (12)	C8B—N1B—C9B—C11B	67.79 (13)
C8A—N1A—C9A—C10A	−48.11 (13)	C8B—N1B—C9B—C10B	−53.48 (14)
C8A—N1A—C9A—C12A	−167.23 (10)	C8B—N1B—C9B—C12B	−172.11 (10)
C7A—N1A—C9A—C12A	32.13 (13)	C7B—N1B—C9B—C12B	11.13 (14)
C8A—C1A—C2A—C3A	178.57 (11)	C8B—C1B—C2B—C3B	176.12 (11)
C6A—C1A—C2A—C3A	−1.08 (17)	C6B—C1B—C2B—C3B	−0.36 (17)
C2A—C1A—C8A—O1A	4.84 (19)	C2B—C1B—C8B—O1B	5.40 (19)
C2A—C1A—C8A—N1A	−175.05 (11)	C2B—C1B—C8B—N1B	−174.09 (11)
C6A—C1A—C8A—O1A	−175.47 (11)	C6B—C1B—C8B—O1B	−177.77 (11)
C2A—C1A—C6A—C5A	−0.26 (18)	C2B—C1B—C6B—C5B	−0.84 (17)
C2A—C1A—C6A—C7A	178.20 (10)	C2B—C1B—C6B—C7B	176.23 (10)
C8A—C1A—C6A—C5A	−179.98 (11)	C8B—C1B—C6B—C5B	−177.96 (10)
C8A—C1A—C6A—C7A	−1.52 (13)	C8B—C1B—C6B—C7B	−0.89 (12)
C6A—C1A—C8A—N1A	4.64 (12)	C6B—C1B—C8B—N1B	2.75 (12)
C1A—C2A—C3A—C4A	1.77 (18)	C1B—C2B—C3B—C4B	1.09 (18)
C2A—C3A—C4A—C5A	−1.17 (19)	C2B—C3B—C4B—C5B	−0.63 (19)
C3A—C4A—C5A—C6A	−0.21 (19)	C3B—C4B—C5B—C6B	−0.57 (19)
C4A—C5A—C6A—C1A	0.91 (18)	C4B—C5B—C6B—C1B	1.29 (18)
C4A—C5A—C6A—C7A	−177.17 (12)	C4B—C5B—C6B—C7B	−175.08 (12)
C5A—C6A—C7A—N1A	176.34 (12)	C5B—C6B—C7B—N1B	175.55 (12)
C1A—C6A—C7A—N1A	−1.91 (12)	C1B—C6B—C7B—N1B	−1.15 (12)

### Hydrogen-bond geometry (Å, º)

**Table:**

*D*—H···*A*	*D*—H	H···*A*	*D*···*A*	*D*—H···*A*
C7*A*—H2*c*7*A*···O1*A*^i^	0.99	2.51	3.4389 (14)	157
C10*A*—H1*c*10*A*···O1*A*	0.98	2.34	2.9149 (12)	117
C4*B*—H1*c*4*B*···O1*A*^ii^	0.95	2.39	3.3237 (15)	167
C10*B*—H2c1B···O1*B*	0.98	2.42	3.0171 (15)	119

Symmetry codes: (i) *x*−1, *y*, *z*; (ii) *x*−1/2, −*y*+1/2, *z*+1/2.
